# The Bosnian version of the international self-report measure of posttraumatic stress disorder, the Posttraumatic Stress Diagnostic Scale, is reliable and valid in a variety of different adult samples affected by war

**DOI:** 10.1186/1471-244X-5-11

**Published:** 2005-02-23

**Authors:** Steve Powell, Rita Rosner

**Affiliations:** 1 Department of Psychology, Ludwig-Maximilians-University, Leopoldstr. 13, 80802 Munich, Germany

## Abstract

**Background:**

The aim of the present study was to assess the internal consistency and discriminant and convergent validity of the Bosnian version of a self-report measure of posttraumatic stress disorder (PTSD), the Posttraumatic Stress Diagnostic Scale (PTDS). The PTDS yields both a PTSD diagnosis according to the Diagnostic and Statistical Manual of Mental Disorders 4^th ^edition (DSM-IV) and a measure of symptom severity.

**Methods:**

812 people living in Sarajevo or in Banja Luka in Bosnia-Herzegovina, of whom the majority had experienced a high number of traumatic war events, were administered the PTDS and other measures of trauma-related psychopathology. The psychometric properties of the instrument were assessed using Cronbach's alpha and principal components analysis, and its construct validity was assessed via Spearman correlation coefficients with the other instruments.

**Results:**

The PTDS and its subscales demonstrated high internal consistency. The principal components revealed by an exploratory analysis are broadly consistent with the DSM-IV subscales except that they reproduce some previously reported difficulties with the "numbing" items from the avoidance subscale. The construct validity of the PTDS was supported by appropriate correlations with other relevant measures of trauma related psychopathology.

**Conclusion:**

The Bosnian version of the PTDS thus appears to be a time-economic and psychometrically sound measure for screening and assessing current PTSD. This self-report measure awaits further validation by interview methods.

## Background

To obtain a diagnosis of PTSD and an estimation of PTSD severity a wide range of measures either relying on interviews or self-report exist in many languages. However, most of the relevant validation studies for these instruments were carried out for English-language versions [1]. For many languages, validated instruments do not exist. A standard approach in this situation is to translate one of those English-language instruments which are well validated, to carry out a validation study for the translation and to compare the results of the validation study with the studies for the original.

Self-report instruments have several advantages as compared to interview measures. They are relatively economic in terms of administration and demand minimal clinician time. If clinicians are not familiar with psychiatric diagnostic procedures and especially the clinical diagnosis of PTSD, it is more advisable to use a psychometrically sound self-report measure which is less prone to mistakes than interview measures.

A good self-report measure for PTSD should allow a diagnosis of PTSD as well as an estimation of PTSD severity and should conform to the DSM-IV criteria for PTSD [2]. The English version of the Posttraumatic Stress Diagnostic Scale [PTDS; [3]] fulfils these criteria and has been shown to have adequate psychometric properties. The PTDS has been translated into a German version which also has adequate psychometric properties [4]. These two different language versions of the PTDS have been used in numerous studies [e.g. [4-9]]. Table 1 provides an overview over the internal consistency and the test-retest-reliability of the PTDS as published in the literature.

**Table 1:** Internal consistency and test-retest reliability of the PTDS and its DSM-III-R precursor the PSS-SR

**Authors**	**Samples**	**Scales**	**Cronbach's alpha**	**Test-retest reliability**
**Foa, Riggs, Dancu, & Rothbaum (1993) [8]**	44 women (rape and other non-sexual attack)	Total score	.91	.74 after one month (N = 29)
	5 – 6 weeks after the event	Reexperiencing	.78	.66
		Avoidance	.80	.56
		Hyperarousal	.82	.71
**Engelhard et al (2001) [7]**	113 women after miscarriage	Total score	.87	
**Stieglitz, Frommberger, Foa, & Berger (2001) [9]**	152 persons:	Total score	.85 and .86	.60 after six months
	1. time point: a few days after accident	Reexperiencing	.75 and .82	.39
		Avoidance	.56 and .74	.53
	2 time point: 6 months later	Hyperarousal	.75 and .64	.47
**Foa, Cashman, Jaycox, & Perry (1997) [3]**	284 victims of various traumatic experiences	Total score	.92	.83 (approx 2 weeks later)
		Reexperiencing	.78	.77
		Avoidance	.84	.81
		Hyperarousal	.84	.85
				Kappa = .74 for PTSD diagnosis

In terms of convergent validity, Foa, Riggs, Dancu, and Rothbaum [8] compared Posttraumatic Symptom Scale scores (PSS-SR; the DSM-III-R version of the PTDS) with the diagnosis obtained by administering the Structured Clinical Interview for the DSM-III-R [SCID;[10]]. 86 % of the participants with a PTSD diagnosis according to DSM-III-R criteria were correctly identified with the self-report instrument. The sensitivity was 62% and the specificity 100%. The DSM-IV version of the PTDS achieved a sensitivity of .89 and a specificity of .75. Percentage agreement between SCID and PTDS diagnosis was 82 % and kappa was .65. Overall, the criterion validity of the PTDS with respect to SCID was encouraging. Table 2 provides an overview of convergent and divergent validity for the PTDS and some other self-report measures of trauma related psychopathology.

**Table 2:** Convergent and divergent validity of the PTDS and its DSM-III-R precursor PSS-SR

**Authors**	**PSS/PTDS Scales**	**IES Total score**	**IES Intrusion**	**IES Avoidance **	**BDI**
**Foa et al. (1993) [8]**	PSS Total score		.81	.53	.80
	Reexperiencing		.81	.47	.66
	Avoidance		.71	.52	.73
	Hyperarousal		.70	.45	.75
**Stieglitz et al. (2001) [9]**	PSS Total score		.67 & .65	.61 & .57	.61 only at first measurement (a few days after the accident)
	Reexperiencing		.63 & .59	.53 & .47	.45
	Avoidance		.56 & .55	.50 & .51	.50
	Hyperarousal		.52 & .49	.47 & .45	.60
**Foa et al. (1997) [3]**	Total score	.78	.80	.66	.79
	Reexperiencing	.68	.77	.51	.67
	Avoidance	.75	.72	.69	.77
	Hyperarousal	.70	.74	.58	.73

The symptom items of the PTDS, which reflect more or less verbatim the corresponding items in the DSM-IV criteria, in empirical studies do not necessarily fall into the three groups explicit in DSM-III-R and DSM-IV. The results of a number of factor-analytic studies suggest that the avoidance symptoms load on two separate factors [11-13]. One factor captures wilful and effortful avoidance and the other factor captures involuntary strategies of "shutting down" the emotional system when effortful strategies fail, which thus may load together on the same factor as hyperarousal symptoms. This issue is to be borne in mind when examining the structure of instruments intended to measure PTSD symptoms according to DSM-IV.

Because of the many advantages of the PTDS we decided to use it for estimating rates of PTSD in a series of studies in different samples of war-traumatized inhabitants of Sarajevo and Banja Luka, Bosnia and Herzegovina. The results of these studies have been published elsewhere or are still in the process of being published [14-16].

The PTDS had to our knowledge never been used before in the area of former Yugoslavia; instead, many studies have used similar but more or less ad-hoc constructed checklist versions of the DSM-IV criteria. The introduction of the PTDS would therefore mean providing clinicians and researchers with a sound Bosnian version of an internationally accepted PTSD self-rating instrument. The goal of this paper is to report first results of the psychometric evaluation of the Bosnian PTDS.

## Methods

### Diagnostic assessment

Although all applied measures are questionnaires, not all subjects proved literate enough to complete them on their own. Therefore in some cases the interviewers had to read some of the questions to them and sometimes to reread or reformulate the questions. Thus the administration deviated slightly from the standard procedures.

The instrument under assessment was the *Posttraumatic Stress Diagnostic Scale *[3,17] which allows, as mentioned before, a diagnosis of PTSD as well as an estimation of symptom severity. The PTDS consists of four parts. Part 1 has 12 items in the original and asks about possible traumatic events (A1 criterion of DSM-IV). In part 2 the time of occurrence of the "most upsetting" event, together with the respondent's assessment of whether the event was life-threatening and whether it was accompanied by feelings of helplessness and intense fear are all evaluated (A2-criterion). Part 3 asks about symptoms of reexperiencing (5 items; criterion B), avoidance (7 items, criterion C), and arousal (5 items, criterion D). Part 4 explores the duration of the disturbance (criterion E) and the consequences of the symptomatology for important areas of functioning (criterion F). Since the original PTDS was designed for a civilian population in times of peace we replaced part 1 with a checklist of traumatic events specific to the war in Bosnia and Herzegovina 1992–5, the Checklist of War Related Experiences, CWE, the items of which are reproduced in Appendix 1. (The checklist also included other significant life events relevant to life in post-war Bosnia-Herzegovina. As these items are not relevant to this study, they are not discussed here.)

To obtain a Bosnian version we applied the procedures suggested by Vijver and Hambleton for the translations of psychological assessment measures [18]. That is, we performed an alternating procedure of translations and back-translations until no significant differences could be detected. In a second step we field-tested the resulting pilot versions to further check the appropriateness of the wording to the Bosnian language and the cultural context. The resulting modifications were then back-translated again.

The **Impact of Event Scale **[IES; [19]] is a questionnaire which assesses the frequency of intrusion and avoidance phenomena as a consequence of experiencing a particular event. In the more than 20 years since its publication it has very frequently been used to diagnose PTSD; however, that is neither the intended nor an appropriate use for it. The IES consists of 15 items each to be answered on a four-point scale assessing the frequency of the occurrence of stress reactions in the preceding week (0 = not at all; 1 = occasionally; 3 = sometimes; 5 = frequently). This means that total scores for the IES range between 0 and 75, with higher scores indicating more frequent intrusion and avoidance reactions. The IES has been applied in nearly every kind of traumatisation [for an overview, see [20]] and has been translated into many languages. The IES is one the most frequently used traumatic stress questionnaires internationally. The version used in the present study was almost identical to one which has been used in other studies in the region during and after the war and which has since been subject to a validation study [21] and found to have satisfactory factor structure and reliability.

**The Symptom Checklist-90-R **[SCL-90-R; [22]] is a 90 item self report questionnaire for measuring subjective psychological and somatic stress in the preceding seven days. Like the IES, the SCL-90-R is used widely internationally and has been used in a large number of research projects in a very wide variety of applications [for an overview, see [23]]. The SCL-90-R consists of nine scales and three global indices, of which the GSI, the Global Severity Index, is the most widely used.

#### Beck Depression Inventory (BDI)

The Beck Depression Inventory [BDI; [24]] is probably the best documented self-report method of measuring the intensity of depression [25,26]. By 1998 more than 2000 studies had been published using the BDI [27]. The current, revised, version consists of 21 items whose scores vary between 0 and 3 [24]. Zero indicates that the symptom is not present whereas three indicates the most extreme level of symptoms. Clients are instructed to report on how they felt in the preceding seven days.

### Samples

The following data was collected between February 1998 and October 1999 in Sarajevo, Banja Luka and Prijedor, which are all in Bosnia-Herzegovina. Sarajevo is in the Federation of Bosnia and Herzegovina, namely that part of Bosnia and Herzegovina which has a predominantly Muslim and Catholic population, and Banja Luka and Prijedor are in the other part, the Republika Srpska, which is predominantly Serbian Orthodox. The samples were stratified by age and sex. The number of years of schooling was also recorded. All subjects participated voluntarily and gave fully informed consent. Table 3 shows sampling procedures, region, and numbers for each sub-sample included in the following analysis. Table 4 provides a description of the demographics.

**Table 3:** Overview of samples used

**Sample**	**Region**	**Sampling procedure**	**N**
A 1998	Sarajevo	randomised via maps of Sarajevo area	98
B 1998	Sarajevo	admission to psychological treatment	114
C 1998	Sarajevo	admission to medical treatment	99
D 1999	Sarajevo	randomly selected repatriates to B&H from lists held by local councils	103
E 1999	Sarajevo	randomly selected displaced or formerly displaced persons from lists held by local councils	97
F 1999	Banja Luka	randomly selected subjects who stayed in the Banja Luka throughout the war, selected via maps of area	100
G 1999	Banja Luka	randomly selected returned displaced persons, selected from lists of residents	100
H 1999	Prijedor	randomly selected from lists of residents in collective centres	100

**Table 4:** Sample description

	N	Minimum	Maximum	Mean	Std. Deviation
years of education	809	8.00	16.00	11.72	2.50439
age	812	16.00	68.00	37.89	13.78230
		N	%	Missing	
sex	female	426	52.5 %		
	male	386	47.5 %		
	Total	812	100.0 %	0	
employment status	unemployed or waiting list	178	21.9 %		
	other (housewife, student)	360	44.3 %		
	employed	274	33.7 %		
	Total	812	100.0 %	0	
family status	single	363	44.8 %		
	married or long-term relationship	447	55.2 %		
	Total	810	100.0%	2	
	Other	70	8.7 %		
religion	Islam	383	47.3 %		
	Catholicism	45	5.6 %		
	Orthodox	311	38.4 %		
	Total	809	100.0 %	3	

In total 812 persons participated. Inclusion criteria for all were a) age between 16 and 65, b) not suffering from a psychotic disorder and c) literate enough to answer the questionnaires with help. All subjects completed the PTDS and the SCL-90-R; therefore correlations for these subscales are based on the data of all the subjects. However for reasons of economy, in 1999 the full package of questionnaires including the BDI and IES were only administered to a random selection of participants in only the two Sarajevo sub-samples. All other participants in 1999 only answered a smaller package of questionnaires including the PTDS. Correlations between the PTDS and BDI and IES are therefore based on a smaller dataset.

In 20 cases an entire instrument was missing, as detailed in table 5. In the remaining cases, the number of individual missing values for individual items was small (much less than 5%), so it was deemed acceptable to form the total scores for the scales simply by multiplying the mean item score for each individual, allowing for any missing items, by the total number of items on each scale. So in the case of the inter-scale correlations the Ns are merely reduced by the number of completely missing questionnaires. In the case of the reliability analyses for the subscales of the PTDS, instruments with any missing items on the scale in question were excluded from the analyses, in each case slightly reducing the Ns.

**Table 5:** Details of which instruments were given to which sub-samples

	BDI			IES			PTDS		SCL		
	not given	missing	available	not given	missing	available	missing	available	missing	available	total

1998 samples, Sarajevo											
non-displaced random sample			98		2	96	1	97	1	97	98
non-displaced medical treatment		1	98		4	95	5	94	1	98	99
non-displaced psychological treatment			114		1	113		114		114	114
1999 samples, Sarajevo											
returnees from outside Former Yugoslavia	40		64	40		62	1	103	2	102	104
displaced or former displaced	21		76	21	1	75		97		97	97
1999 samples, Banja Luka and Prijedor											
Banja Luka displaced or former displaced	100			100				100		100	100
Banja Luka non-displaced	100			100				100		100	100
Prijedor displaced in camps	100			100				100		100	100
Table Total	361	1	450	361	8	441	7	805	4	808	812

### Interviewers

The medical and psychological samples were assessed through a total of 15 experienced counsellors/therapists, who were working at a variety of clinics and counselling centres in Sarajevo. All other samples were assessed by pairs of final year and third year students of Psychology at Sarajevo University and Banja Luka University. All interviewers were trained in the use of the questionnaires. Two pilot studies were performed to insure the appropriate use of the assessment. During the studies constant supervision for all interviewers was provided.

### Statistical analysis

To obtain an estimation of internal consistency Cronbach's alpha was calculated for the total scores and the subscales of the PTDS. Convergent and divergent validity were estimated by using Spearman correlations between the scales. Spearman correlations were used because most of the distributions were not normal. For the principal components analysis, oblimin oblique rotation was used.

## Results and discussion

The standardised Cronbach's alphas for the Bosnian PTDS were .93 for the total symptom score, .89 for the reexperiencing subscale, .84 for the avoidance subscale and .84 for the arousal subscale. The results correspond well with other published results.

The Spearman's correlations between the total scale and the subscales were all quite high at .89, .93 and .87 for re-experiencing, avoidance and hyperarousal respectively; re-experiencing correlated .74 and .67 with avoidance and hyperarousal; and the correlation between avoidance and hyperarousal was .72.

The item characteristics for the symptom items and subscale totals are shown in table 6. The characteristics are acceptable, with the lowest standard deviation being .77 for the item about not being able to remember details, which also had the lowest mean (.36 on a scale of 0 to 4).

**Table 6:** Item characteristics of the PTSD symptom items of the Bosnian PTDS

	sex					
	female		male		total	

	Mean	Standard Deviation	Mean	Standard Deviation	Mean	Standard Deviation

B1 intrusions	1.00	1.12	.76	1.00	.89	1.07
B2 bad dreams	.73	1.02	.57	.92	.65	.98
B3 reexperiencing	.70	.99	.53	.86	.62	.94
B4 upset after remembering	1.14	1.07	.89	1.01	1.02	1.04
B5 physical reaction after remembering	.95	1.09	.62	.92	.79	1.02
C1 attempt not to think about it	1.14	1.16	.83	1.06	.99	1.12
C2 avoiding places people	.86	1.13	.65	1.03	.76	1.09
C3 not being able to remember details	.40	.80	.33	.74	.36	.77
C4 less interest in activities	.66	.96	.51	.89	.59	.93
C5 detachment estrangement	.65	1.00	.50	.93	.58	.97
C6 restricted affect	.81	1.07	.50	.87	.66	.99
C7 foreshortened future	.79	1.08	.58	.95	.69	1.02
D1 difficulty falling or staying asleep	.92	1.11	.63	.96	.78	1.05
D2 irritability	.70	.94	.55	.88	.62	.92
D3 difficulty concentrating	.92	1.02	.68	.92	.80	.98
D4 hypervigilance	.55	.88	.40	.77	.48	.83
D5 exaggerated startle response	.75	.99	.42	.81	.60	.93
total score on subscale b (reexperiencing)	4.53	4.43	3.36	3.94	3.97	4.24
total score on subscale c (avoidance)	5.32	5.02	3.89	4.79	4.64	4.96
total score on subscale d (arousal)	3.82	3.77	2.67	3.44	3.28	3.66
total score on all symptom subscales	13.66	11.73	9.93	10.84	11.88	11.46

The items were scored on a scale of 0 (not at all or once a month) to 4 (5 or more times a week /almost always).

The items from the symptom subscales were submitted to a principal components analysis with oblimin oblique rotation. Factors with eigenvalues greater than 1 were retained. Items were considered as belonging to a factor if their loadings on that factor were above 0.4. (see table 7). The first solution had three factors explaining a total of 61.41% of the variance and was deemed to be satisfactory, so that no further solutions were sought. The first factor, which explains 47.64% of the variance, was labelled Arousal / Numbing. It contains all the items from the DSM-IV arousal scale and three DSM-IV avoidance items, two of which (detachment/estrangement and restricted affect) are also associated with numbing [11]. The second factor, explaining 7.85% of the variance, was labelled Intrusion and includes all the items from the DSM-IV intrusion scale together with one item ("attempting not to think about it") from the DSM-IV avoidance scale. The third factor, which explains 5.92% of the variance, was labelled Avoidance. It contains all the items from the DSM-IV avoidance scale except for two items which load on Arousal/Numbing. Every item loaded on at least one factor and only two items loaded on more than one factor (the item "attempt not to think about it" loaded on the Intrusion and Avoidance factors, and the item "detachment, estrangement" loaded on the Arousal/Numbing and Avoidance factors).

**Table 7:** Rotated factor pattern of the PTSD symptom items of the Bosnian PTDS

	Loadings		
Symptom	Factor 1: Arousal / Numbing	Factor 2: Intrusion	Factor 3: Avoidance

b1 intrusions	.031	**-.824**	.050
b2 bad dreams	.127	**-.779**	-.049
b3 reexperiencing	.189	**-.704**	-.047
b4 upset after remembering	-.042	**-.830**	.079
b5 physical reaction after remembering	.084	**-.729**	.074
c1 attempt not to think about it	-.163	**-.480**	**.544**
c2 avoiding places people	-.080	-.256	**.666**
c3 not being able to remember details	.103	.014	**.649**
c4 less interest in activities	.209	-.105	**.511**
c5 detachment, estrangement	**.567**	.089	**.438**
c6 restricted affect	**.523**	-.002	.397
c7 foreshortened future	**.596**	.056	.326
d1 difficulty falling or staying asleep	**.460**	-.361	.063
d2 irritability	**.652**	-.199	-.031
d3 difficulty concentrating	**.732**	-.123	-.017
d4 hypervigilance	**.753**	-.072	-.122
d5 exaggerated startle response	**.746**	-.065	-.003

Factor loadings greater than 0.40 are shown in bold underline.

In short, the three DSM-IV scales can be broadly identified, except that three DSM-IV avoidance items including two of the somewhat contentious numbing items load on the arousal scale, which replicates well the findings reported above [11-13].

Table 8 provides the correlations between the various other measures of psychopathology and the Bosnian PTDS. With samples of this size, correlations even as small as approximately .1 are significant, so all the correlations are highly significant and thus the significances are not reported here.

**Table 8:** Convergent and divergent validity of the Bosnian PTDS

		**IES Total**	**IES Intrusion**	**IES Avoidance**	**BDI**	**SCL GSI**
PTDS Total	Spearman's rho	.709	.703	.619	.622	.568
	N	439	438	439	444	802
Reexperiencing	Spearman's rho	.634	.687	.491	.485	.437
	N	439	438	439	444	802
Avoidance	Spearman's rho	.651	.603	.610	.581	.515
	N	439	438	439	444	802
Hyperarousal	Spearman's rho	.573	.574	.493	.596	.595
	N	439	438	439	445	803

The correlations between the PTDS and the IES are somewhat lower than in the two American publications, closer to those in the German article. Re-experiencing on the PTDS correlates higher with intrusion than with avoidance on the IES, and avoidance on the PTDS correlates higher with avoidance on the IES than with intrusion on the IES, all of which are desirable results in that they support construct validity. The correlations between the re-experiencing and avoidance scales of the IES and the avoidance scale of the PTDS are quite similar, possibly indicating weak specificity of the latter, which was however also the case for all except the oldest of the three previous studies.

The correlation between the BDI total and the PTDS/PSS total is high, as reported in the literature. In fact the Bosnian version seems to differentiate a little better between PTSD and depression than do the American and German versions; nevertheless the specificity is still quite weak.

In the same way there are also quite high correlations with the SCL-90-R. Although the Bosnian version of the BDI and SCL have also not been adequately validated before, validating one new instrument against other instruments which are also not validated is not a meaningless affair but on the contrary the only possible procedure in a situation such as the one we (and our local and international researcher colleagues) found ourselves in, namely that very few world-standard instruments existed. If one does find, as we did, inter-instrument correlations similar to those for the corresponding instruments in other languages then that provides at least some provisional evidence for the psychometric quality and construct validity of all of those instruments.

One of the main uses of the PTDS is to provide a PTSD diagnosis in an economical way. As the PTDS assesses in questionnaire form all the information necessary for the diagnosis according to DSM-IV, the PTDS prevalences can be easily calculated and are in fact 24.72% for the whole sample, 31.37% for women and 17.40% for men.

The most important factor which restricts the interpretation of these results is that the PTDS was not compared with clinical interview, which would have been standard procedure in this kind of study. However, when we began the study there was no suitable validated interview available in the Bosnian language, which meant that we would have had to translate and extensively validate such an interview ourselves, and again we would have run into the problem of validating the interview against instruments which had also not been validated at that time. It also should be stressed that this study says very little about the cultural or contextual validity of the instrument or the construct PTSD which it is intended to measure.

On the other hand, the samples are quite large and taken together quite heterogeneous, and the selection methodologies in each case provided a reasonable approximation to randomness, so that all in all the data can be considered to be of good quality.

## Conclusion

In conclusion it can be said that the psychometric properties of the Bosnian version of the PTDS are as good as those published for other languages. The internal consistencies are at least as good and the Bosnian version appears even to distinguish a little better than the American and German versions between PTSD as measured by the IES and depression as measured by the BDI. The principal components revealed by an exploratory analysis are broadly consistent with the DSM-IV subscales except that they reproduce some previously reported difficulties with the "numbing" items from the avoidance subscale; this issue might explain the poor specificity of the avoidance scale with respect to the IES subscales. None of the analyses revealed anything unusual or indicated problems either with the translation or with the application of the concepts inherent in the instrument to the post-war Bosnian population, all of which indicates that the Bosnian PTDS can be given the green light for further application in the future. Yet our results are only a necessary first step in the validation of the applied measures; a comparison with a validated translation of a Bosnian interview measure for PTSD still needs to be done.

## Competing interests

The author(s) declare that they have no competing interests.

## Authors' contributions

RR participated in the design of the study, and drafted the manuscript.

SP carried out the actual study and performed the statistical analysis.

Both authors worked on and approved the final manuscript.

## Appendix 1

### The war traumatic event items of the Checklist of War Events (which replaces the standard traumatic event checklist in the PTDS)

group 0: injury to self

Were you severely injured during the war?

group 1: sexual violence to self

Were you raped or sexually assaulted during the war?

During the war, were you sexually assaulted by a member of your close family who had been forced to do that?

During the war, were you sexually assaulted by a member of your close family who was not forced to do that?

group 2: torture to self

Were you tortured during the war?

group 3: other threat to self

During the war, were you in a situation in which you strongly believed you would be severely injured or killed?

During the war, did a bullet come so close to you that you could have been severely injured or killed?

During the war, did a bomb or grenade explode so close to you that you could have been severely injured or killed?

During the war, did anyone threaten to kill you or severely injure you?

Were you captured or held in a detention camp during the war?

During the war, were you without food or water for so long that you strongly believed you would die?

During the war, were you so cold that you strongly believed you would die?

During the war, did you stay in a cellar longer than 3 weeks without a break?

During the war, were you assaulted in a non-sexual way by a member of your close family who had been forced to do that?

During the war, were you assaulted in a non-sexual way by a member of your close family who had not been forced to do?

Were you in the army during the war?

During the war, were you seriously ill because of the war (e.g. heart attack)

group 4: witnessed: loved ones

Did you eyewitness a loved one being killed during the war?

Did you see dead body of a loved one who had been killed in the war? (excluding funerals)

Did you see a loved one being tortured or physically assaulted during the war?

Did you see a loved one being sexually assaulted during the war?

Did you touch a loved one who had been killed or wounded in the war?

During the war, did you see a loved one who was severely injured before he/she received medical help?

group 5: witnessed: others

Did you eyewitness somebody being killed (not a loved one) in the war?

Did you see the body of a person (but not a loved one) who had been killed in the war? (excluding funerals)

Did you see someone being tortured or physically assaulted during the war (but not a loved one)?

Did you see someone being sexually assaulted during the war (but not a loved one)?

Did you touch someone (but not a loved one) who had been killed or wounded in the war?

During the war, did you see a severely injured person (not a loved one) before they received medical help?

group 6: losses, nuclear family

Was your father killed in the war?

Was your mother killed in the war?

Was your spouse killed in the war?

Was a child of yours killed in the war?

Was a brother or sister of yours killed in the war?

group 7: losses, other loved ones

Was a close relative of yours killed in the war?

Was a close friend of yours killed in the war?

group 8: threat, violence, injury to loved ones

Was a loved one in the army during the war?

Was a loved one severely injured in the war?

Was a loved one raped or sexually assaulted in the war?

Was a loved one tortured in the war?

Was a loved one captured or held in a concentration camp during the war?

During the war, was a loved one seriously ill (e.g. cancer or heart attack) or had some chronic health problem?

group 9: other war events

Other traumatic event since 1991 due to war: 1

Other traumatic event since 1991 due to war: 2

Other traumatic event since 1991 due to war: 3

group 10: other events since 1991 not related to war

Did a loved one die in the war for reasons unrelated with the war?

(Other stressful and traumatic events since 1991 and unrelated to the war)

## Pre-publication history

The pre-publication history for this paper can be accessed here:



## References

[B1] Schnurr PP, Friedman MJ, Bernardy NC (2002). Research on posttraumatic stress disorder: Epidemiology, pathophysiology, and assessment. J Clin Psychol.

[B2] American Psychiatric Association (1994). Diagnostic and statistical manual of mental disorders 4th edition (DSM-IV).

[B3] Foa EB, Cashman L, Jaycox L, Perry K (1997). The validation of a self-report measure of posttraumatic stress disorder: The Posttraumatic Diagnostic Scale. Psychol Assess.

[B4] Steil R, Ehlers A Diagnostische Skala für die Posttraumatische Belastungsstörung Göttingen: Hogrefe.

[B5] Clohessy S, Ehlers A (1999). PTSD symptoms, response to intrusive memories and coping in ambulance service workers. Br J Clin Psychol.

[B6] Ehlers A, Mayou RA, Bryant B (1998). Psychological predictors of chronic posttraumatic stress disorder after motor vehicle accidents. J Abnorm Psychol.

[B7] Engelhard IM, van den Hout MA, Arntz A (2001). Posttraumatic stress disorder after pregnancy loss. Gen Hosp Psychiatry.

[B8] Foa EB, Riggs DS, Dancu CV, Rothbaum BO (1993). Reliability and validity of a brief instrument for assessing posttraumatic stress disorder. J Trauma Stress.

[B9] Stieglitz RD, Frommberger U, Foa EB, Berger M (2001). Evaluation of the German version of the PTSD Symptom Scale (PSS). Psychopathology.

[B10] Spitzer RL, Williams JBW, Gibbon M, First MB (1990). Structured Clinical Interview for DSM-III-R-Patient ed (with Psychotic Screen; SCID-P).

[B11] Foa EB, Riggs D, Gershuny B (1995). Arousal, numbing, and intrusion: Symptom structure of PTSD following assault. Am J Psychiatry.

[B12] King LA, King DW (1994). Latent structure of the Mississippi Scale for Combat-Related Posttraumatic Stress Disorder: Three studies in reliability and validity. J Consult Clin Psychol.

[B13] Taylor S, Kuch K, Koch WJ, Crockett DJ, Passey G (1998). The structure of Posttraumatic stress symptoms. J Abnorm Psychol.

[B14] Rosner R, Powell S, Butollo W (2003). Posttraumatic Stress Disorder after the siege of Sarajevo. J Clin Psychol.

[B15] Powell S, Rosner R, Butollo W, Tedeschi RG, Calloun LG (2003). Posttraumatic growth after war: A study of former refugees and displaced people in Sarajevo. J Clin Psychol.

[B16] Rosner R, Powell S, Butollo W (2002). Why do people in Bosnia-Herzegovina go into treatment. The role of Posttraumatic Stress Disorder in psychotherapy service utilization. European Psychotherapy.

[B17] Foa EB (1996). Posttraumatic Stress Diagnostic Scale.

[B18] Vijver F, Hambleton RK (1996). Translating Tests: Some practical guidelines. European Psychologist.

[B19] Horowitz M, Wilner N, Alvarez W (1979). Impact of Event Scale: A measure of subjective stress. Psychosom Med.

[B20] Joseph S (2000). Psychometric Evaluation of Horowitz's Impact of Event Scale: A Review. J Trauma Stress.

[B21] Mooren TTM (2001). The impact of war.

[B22] Derogatis LR (1977). SCL-90-R: Administration, Scoring and Procedures Manual for the R (evised) Version.

[B23] Franke G, Stäcker KH (1995). Reliabilität und Validität der Symptom-Check-Liste (SCL-90-R; Derogatis, 1986) bei Standardreihenfolge versus inhaltshomogener Itemblockbildung. Diagnostica.

[B24] Beck AT (1978). Depression Inventory.

[B25] Endler NS, Macrodimitris SD, Kocovski NL (2000). Depression: The complexity of self-report measures. J Applied Biobehavioral Res.

[B26] Naughton MJ, Wiklund I (1993). A critical review of dimension-specific measures of health-related quality of life in cross-cultural research. Qual Life Res.

[B27] Richter P, Werner J, Heerlein A, Kraus A, Sauer H (1998). On the validity of the Beck Depression Inventory: A review. Psychopathology.

